# Lung Segmentation on High-Resolution Computerized Tomography Images Using Deep Learning: A Preliminary Step for Radiomics Studies

**DOI:** 10.3390/jimaging6110125

**Published:** 2020-11-19

**Authors:** Albert Comelli, Claudia Coronnello, Navdeep Dahiya, Viviana Benfante, Stefano Palmucci, Antonio Basile, Carlo Vancheri, Giorgio Russo, Anthony Yezzi, Alessandro Stefano

**Affiliations:** 1 Ri.MED Foundation, 90133 Palermo, Italy; ccoronnello@fondazionerimed.com; 2 Institute of Molecular Bioimaging and Physiology, National Research Council (IBFM-CNR), 90015 Cefalù, Italy; viviana.benfante@ibfm.cnr.it (V.B.); giorgio.russo@ibfm.cnr.it (G.R.); alessandro.stefano@ibfm.cnr.it (A.S.); 3 Department of Electrical and Computer Engineering, Georgia Institute of Technology, Atlanta, GA 30332, USA; ndahiya3@gatech.edu (N.D.); anthony.yezzi@ece.gatech.edu (A.Y.); 4 Department of Medical Surgical Sciences and Advanced Technologies, Radiology Unit I, University Hospital “Policlinico-Vittorio Emanuele”, 95123 Catania, Italy; spalmucci@sirm.org (S.P.); basile.antonello73@gmail.com (A.B.); 5 Regional Referral Centre for Rare Lung Diseases, A.O.U. Policlinico-Vittorio Emanuele, University of Catania, 95123 Catania, Italy; vancheri@unict.it

**Keywords:** idiopathic pulmonary fibrosis, deep learning, lung segmentation, high resolution computed tomography, radiomics, U-Net, E-Net

## Abstract

Background: The aim of this work is to identify an automatic, accurate, and fast deep learning segmentation approach, applied to the parenchyma, using a very small dataset of high-resolution computed tomography images of patients with idiopathic pulmonary fibrosis. In this way, we aim to enhance the methodology performed by healthcare operators in radiomics studies where operator-independent segmentation methods must be used to correctly identify the target and, consequently, the texture-based prediction model. Methods: Two deep learning models were investigated: (i) U-Net, already used in many biomedical image segmentation tasks, and (ii) E-Net, used for image segmentation tasks in self-driving cars, where hardware availability is limited and accurate segmentation is critical for user safety. Our small image dataset is composed of 42 studies of patients with idiopathic pulmonary fibrosis, of which only 32 were used for the training phase. We compared the performance of the two models in terms of the similarity of their segmentation outcome with the gold standard and in terms of their resources’ requirements. Results: E-Net can be used to obtain accurate (dice similarity coefficient = 95.90%), fast (20.32 s), and clinically acceptable segmentation of the lung region. Conclusions: We demonstrated that deep learning models can be efficiently applied to rapidly segment and quantify the parenchyma of patients with pulmonary fibrosis, without any radiologist supervision, in order to produce user-independent results.

## 1. Introduction

Biomedical images are a huge source of data useful to feed diagnostic tools with increasing performance in revealing pathologies. Through radiomics tools, researchers are able to retrieve informative features from images. The correct identification of the anatomical region of interest is crucial to avoid errors in the extraction of features and in their associations with the studied target. For this reason, there is a growing interest in the use of image-segmentation strategies to reveal diseases applying radiomics [1,2,3]. Focusing on the target of interest limits the noise in texture-based feature extraction. Nevertheless, the segmentation step is still a challenging issue in the medical image analysis research area [4,5]. The most common technique used to obtain lung images of patients with idiopathic pulmonary fibrosis (IPF) is high-resolution computed tomography (HRCT). IPF patient HRCT scans show fibrotic regions, honeycombing, extensive patchy ground-glass regions with or without consolidations, and the presence of pleural fluid [6]. These characteristics make it difficult to develop an automated lung segmentation algorithm. In particular, some challenges have yet to be overcome:

- For HRCT studies, the analysis of hundreds of slices is required. Using a supervised segmentation algorithm, such as in our previous works [7,8,9], both visual inspection and manual correction are time-consuming.
- For radiomics analysis, accurate and user-independent segmentations are mandatory to correctly identify the texture-based prediction model.
- Artificial intelligence approaches are still far from being widely applied in clinical practice, mainly due to the requirement of large amounts of labelled training data.

To address these issues, we propose a segmentation workflow based on deep learning (DL) using a small dataset of images. DL methods are more efficient than classical statistical approaches in unravelling enormous amounts of data, and they have been recently applied to biomedical image segmentation tasks, showing high performance in image classification and the segmentation of several anatomic districts [10,11,12,13,14]. A DL-based workflow is defined by the following: (i) the training/testing data set (in our case biomedical images, e.g., HRCT); (ii) the outcome variable (in our case, image masks defining the segmentation output); (iii) training and testing algorithm; and (iv) a performance evaluator algorithm. With respect to machine learning algorithms, DL requires more data and it is less transparent, making it difficult in clinical practice to retrieve from the trained algorithms some medical insights. Several categories of DL have been developed, including fully convolutional networks [15], encoder–decoder networks [16], multi-scale and pyramid network-based models [17,18,19], attention-based models [20], recurrent neural network-based models [21], and those based on generative and adversarial training [22,23]. For the lung segmentation task, several deep learning-based methods have been proposed (e.g., [6,11,24,25,26]). The interested reader is encouraged to refer to comprehensive reviews [27,28].

In our study, we focused on the development of DL models that provide accurate and fast segmentation results after being trained with a small image dataset. Specifically, our dataset was composed of 42 high-resolution CT (HRCT) studies of IPF patients, of which only 32 were used for the training phase. One of the DL algorithms successfully applied in the biomedical image segmentation task is the convolutional neural network-based algorithm U-Net [29]. We explored the efficacy of an enhanced U-NET model for the task of lung segmentation in comparison with another model, namely the efficient neural network (E-Net) [30]. E-Net was developed for image segmentation and recognition in self-driving car applications. It is designed to be used in a limited hardware solution, and as a consequence, we wanted to take advantage of its fast training and low training data requirements. To our knowledge, this model has been never applied to biomedical image segmentation before. In addition, we implemented the Tversky loss function into the training process [31], applied a suitable data augmentation technique, adapted the original DL models, and used the k-fold strategy. In this way, we aim to enhance the methodology performed by healthcare operators in radiomics analyses where an operator-independent segmentation method must be used to extract target regions (in our case, the lungs) and, consequently, to obtain operator-independent results. In the following, to evaluate the performance of U-Net and E-Net algorithms, we considered the accuracy of the segmentation, the training time, the hardware requirements, and the amount of training data required.

## 2. Materials and Methods

### 2.1. Data and Hardware Setup

Because our aim was to identify an automatic, accurate, and fast segmentation method of the parenchyma without any radiologist supervision, as in our previous works [7,8,9], we used the same dataset of images performed in our radiology department. Specifically, a retrospective analysis of our interstitial lung disease database identified a total of 210 patients who had received a multidisciplinary team diagnosis of IPF according to 2011 ATS/ERS/JRS/ALAT IPF guidelines [32]. Only patients with an unenhanced, supine, volumetric thin-section CT exam (no more than 1.25 mm) performed in our department equipped with Philips CT and GE CT scanners were included in the analysis. A total of 42 consecutive IPF patients fulfilling these criteria were finally included in the analysis. The proposed study was approved by the ethics committee of the Policlinico-Vittorio Emanuele Hospital of Catania (letter number 0039547, protocol QH-IPF, date 5 September 2018). In addition, because IPF affects males more frequently than females, the majority of patients in our study were male. Specifically, 10 out of 42 patients were women, and patient’s ages ranged from 51 to 82 years. Scans were obtained at full inspiration from the apex to the lung base with the patients in the supine position. Thin-section CT images were with sharp kernel imaging reconstruction, contiguous or overlapping images.

Due to the use of a different CT scanner, the whole dataset had different resolutions:

- 11 studies obtained using the Philips CT scanner have a matrix resolution of 720 × 720
- 31 studies obtained using the GE CT scanner have a matrix resolution of 672 × 672.

DL models typically require all inputs to be of the same size. Consequently, we resampled all datasets to the isotropic voxel size of 1 × 1 × 1 mm^3^ with a matrix resolution of 512 × 512 using linear interpolation. Starting from the whole dataset of 42 patient studies, 32 patient studies were used both as training and validation sets using the k-fold strategy (see Section 2.4), while 10 patient studies (not used at all during the training process) were used as a testing set. The grouping was made so that both the sets maintained the same features of the whole dataset (scanner type and patient sex). The ground truth was obtained using a semi-automatic and supervised segmentation method, as described in [7], to discriminate lungs from other structures and to eliminate the trachea. Briefly, digital imaging and communications in medicine (DICOM) images were anonymized using Osirix software [33] before automatic HRCT segmentation was obtained using the region growing algorithm. Voxels within a range of +200 and −1.024 HU were isolated. Successively, the same region growing method was used to eliminate the trachea. Each scan was then segmented using this conventional image processing method and manually corrected by an expert thoracic radiologist to create gold standards. In particular, a 10-year thoracic radiologist (S.P. author) inspected the obtained segmentation and, in the case of coarse anatomical misrecognition or inaccurate segmentation, manually guided the delineation. Specifically, the region growing method was able to detect disease-free lung regions, while some lung regions with high fibrotic areas were not correctly included in the segmentation. Consequently, manual corrections required about 5–10 min for each study. After the supervised validation process, the segmentation masks were then resampled using nearest neighbor interpolation and converted to binary values with 0 for background and 1 for the parenchyma region. We used an in-house processing tool developed in MATLAB^®^ R2016a (The MathWorks, Natick, MA, USA), running on an iMac (3.5 GHz Intel Core i7 processor, 16 GB memory random-access memory; Apple Computer, Cupertino, CA, USA) with Mac Operating System OS X El Capitan. We implemented U-Net, and E-Net networks and Tversky loss (see Section 2.3) using Keras with Tensorflow backend in the open-source programming language Python (www.python.org).

### 2.2. U-Net and E-Net

Several changes were made to the original U-Net architecture to improve segmentation results [29]. All 3 × 3 convolutions were replaced by larger 5 × 5 convolution operators to increase the receptive field of the network. In our experience, 5 × 5 convolutions produced better results in using U-Net for image segmentation tasks. Each convolution was followed by a drop out layer with rate of 10%. Dropout layers help to regularize the network and avoid overfitting. While the original U-Net architecture does not use padding when applying convolution operators, we adopted zero padding to ensure that the size of the output feature map was the same as the input size. The original U-Net had a 2D size of 32 × 32 along with 1024 feature maps at the final layer of the contraction path. In a different way, we used an input size of 512 × 512 with 32 filters on the first contraction path layer, with doubling of feature maps after each max pool and stopping at 256 feature maps and 2D size of 64 × 64. Specifically, we refer to (i) “feature maps” as the output of a convolution layer in DL models highlighting the fact that the input images are converted to outputs that represent interesting hidden features present in the input, and to (ii) “max pool” as the typical operation in convolutional neural networks (CNN) used to down-sample an input representation. This is typically done by selecting only the maximum value from non-overlapping rectangular sub-regions of the input. For example, if we select the maximum from every 2 × 2 sub-region of the input, the output dimensions would be reduced by a factor of two in both height and width.

Concerning E-Net, it was developed for fast inference and high accuracy in augmented reality and automotive scenarios [30]. The E-Net architecture was based on building blocks of residual networks, with each block consisting of 3 convolutional layers. These were a 1 × 1 projection that reduced dimensionality, with a regular convolutional layer and a 1 × 1 expansion along with batch normalization. E-Net adopted several types of convolutions to build an encoder/decoder style image segmentation network. In some layers, E-Net had asymmetric convolutions characterized by separable convolutions with sequences of 5 × 1 and 1 × 5 convolutions. The 5 × 5 convolution had 25 parameters, while the corresponding asymmetric convolution had only 10 parameters to reduce the network size. Finally, the E-Net used a single starting block in addition to several variations of the bottleneck layer. The term “bottleneck” refers to a layer that has been down-sampled several times, and it is used to force the network to learn the most important features present in the input. In this way, the network learns to ignore irrelevant parts of the input data

### 2.3. Loss Function

Several loss functions have been proposed in the literature. The dice similarity coefficient (DSC) is routinely used as loss function in many DL-based medical image segmentation networks, and it measures the overlap between predicted and ground truth segmentation masks. Specifically, it is the harmonic mean of false positives (FPs) and false negatives (FNs) and weighs both equally. To make adjustment of the weights of FPs and FNs, the authors in [31] proposed the Tversky loss based on the Tversky index [34], defined as follows:

(1) $$S\left(P,G;\alpha \;\beta \right)=\frac{\left|P\cap G\right|}{\left|P\cap G\right|+\alpha \left|P\backslash G\right|+\beta \left|G\backslash P\right|}$$

where *P* and *G* are the set of predicted and ground truth labels, respectively, α and β control the magnitude of penalties of FPs and FNs, respectively, and *P*\*G* is the relative complement of *G* on *P*. Using the Tversky index, the Tversky loss is defined as follows:

(2) $$T\left(\alpha \;\beta \right)=\frac{\sum_{i=1}^{N}p_{0i}\;g_{0i}}{\sum_{i=1}^{N}p_{0i}\;g_{0i}+\alpha \sum_{i=1}^{N}p_{0i}\;g_{1i}+\beta \sum_{i=1}^{N}p_{1i}\;g_{0i}}$$

where in the output of the final layer of the network (soft-max layer), *p*_0*i*_ is the probability of voxel *i* being part of the lung, and *p*_1*i*_ is the probability of it belonging to the background. In addition, the ground truth training label *g*_0*i*_ is 1 for lung and 0 for everything else (background), and vice-versa for the label *g*_1*i*_. By adjusting the parameters, α and β, the trade-off can be controlled between FPs and FNs. Setting  α = β = 0.5 leads to the familiar DSC, while setting α + β = 1 leads to a set of $F_{\beta }$ scores; *β*’s larger than 0.5 weight recall higher than precision by placing more emphasis on FNs in the slices with small foreground areas.

### 2.4. Data Training

In typical machine learning and DL approaches, the dataset is divided into three parts, namely training/validation/testing sets. The testing set is also called the hold-out set, which is set aside during the training process and is only used for reporting final results. In addition, if only a limited amount of data is available for the training process, such as in the case of medical image processing, the k-fold cross-validation strategy can be used. The available data are divided into k-folds. One of the folds is then treated as the validation set and the remaining folds combined into the training set. This process is repeated several times using each fold as the validation set and other remaining sets as the training set. In this way, it is possible to get more robust results. For this reason, due to limited amount of data for the training step (32 patient studies), we adopted a five-fold cross-validation strategy by randomly dividing the whole dataset into 5 folds of size of 6 or 8 patients (4 folds of 6 patients, 1 fold of 8 patients). For each network model under consideration, we trained 5 models by combining 4 of the 5 folds into a training set and keeping the remaining fold of 6 or 8 patients as a validation fold. Since all considered network models are 2D models, we extracted individual slices from each study in the training fold and used these individual slices as input to our models. The performance of the k-fold cross-validation strategy was calculated by averaging the results of all 5 validation folds. Finally, the performance metrics for the two DL algorithms were calculated on 10 patient studies (the testing set) not used at all during the k-fold cross-validation strategy.

Data augmentation is a common strategy used to train neural network models, which helps to reduce overfitting, especially in case of limited training data. We also applied this strategy by randomly rotating, translating in both x and y directions, and applying shearing, horizontal flip, and zooming to the input training slices. In total, we used 6 different types of data augmentation techniques. Similarly to the training step, all HRCT slices of each study were used. Additionally, data standardization or normalization is commonly done as a pre-processing step in machine learning, which prevents the weights from becoming too large and helps the models to converge faster and avoid numerical instability.

For each fold, we compute a 2D pixel-wise mean and standard deviation using all training data of that fold. Before being fed into the training pipeline, we subtracted the mean and divided by the pre-computed standard deviation.

We used an initial set of 16 patient studies to experimentally determine the best learning rates. Learning rates of 0.0001 for E-Net model and 0.00001 for U-Net models with Adam optimizer [35] were used. A batch size of 8 slices for all experiments, and α = 0.3 and β = 0.7 for the Tversky loss function were used. During the training process, we allowed each network to train for a maximum of 100 epochs. We used the training loss as a stopping criteria. When the training loss did not decrease for 10 epochs continuously, we stopped the training. Finally, we used an NVIDIA QUADRO P4000 with 8 GB of RAM to train all networks and run inference. Finally, 10 cases were used for the testing step.

### 2.5. Data Analysis

To assess the performance of automatic segmentation, for each clinical case, we computed a set of performance indicators routinely used in literature for shape comparison [36]. Sensitivity, positive predictive value (PPV), dice similarity coefficient (DSC), volume overlap error (VOE), volumetric difference (VD), and average symmetric surface distance (ASSD) were calculated as mean, standard variation (std), and confidence interval (CI). Analysis of variance (ANOVA) on the DSC was used to assess statistical differences among network. Statistical significance was considered for *p*-value ≤ 0.05.

## 3. Results

**Figure 1:**
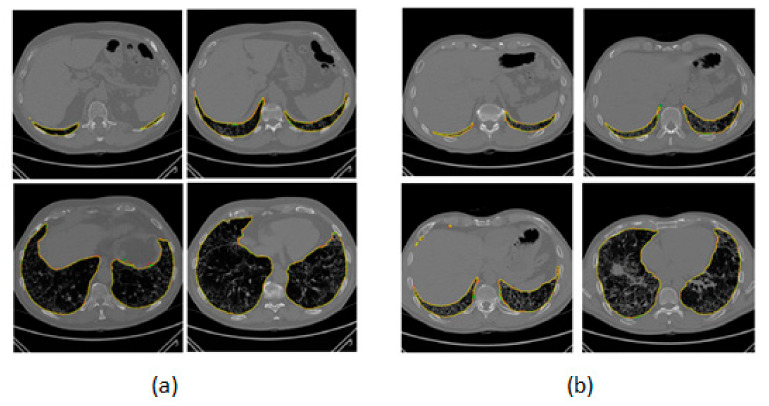
Two examples of lung segmentations (4 slices for both (**a**,**b**) high-resolution computed tomography (HRCT) studies). The manual segmentation (yellow), E-Net (red), and U-Net (green) are superimposed in both (**a**,**b**) images.

**Figure 2:**
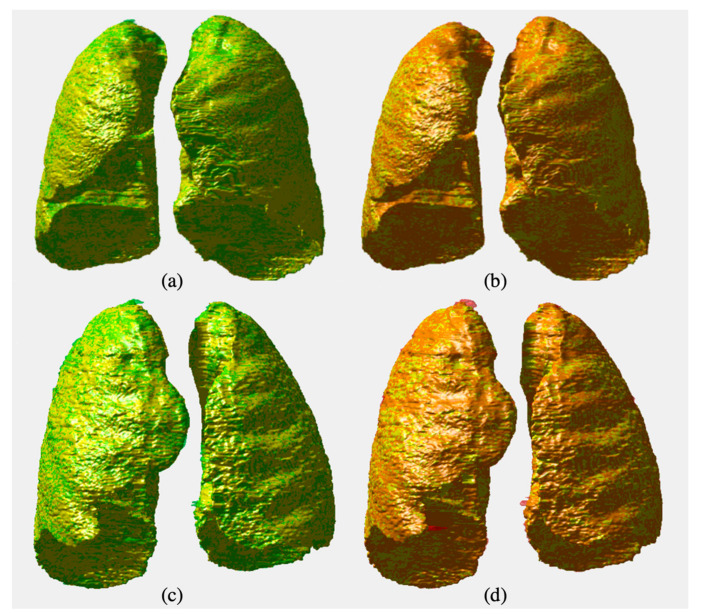
Three-dimensional rendering of two parenchyma delineations. The manual segmentation (yellow), U-Net (green, (**a**,**c**)), and E-Net (red, (**b**,**d**)) are superimposed.

We applied DL methods to obtain the contour of the parenchyma region. Examples of obtained lung segmentations are shown in Figure 1. Additionally, 3D reconstructions of lungs produced using our in-house processing tool developed in MATLAB^®^ R2016a (The MathWorks, Natick, MA, USA) are shown in Figure 2. To emphasize volume intersections, manual and automatic segmentations were superimposed. To evaluate the performance of each method, we cumulatively considered the results related to all the slices of which the HRCT image was composed in order to provide the comparison of the whole three-dimensional shape of the parenchyma with the gold standard. Table 1 shows the performance evaluation in the validation set (32 patient studies using the k-fold strategy, as reported in Section 2.4). Specifically, the E-Net showed a mean DSC of 98.34 ± 1.44%, and a U-Net of 98.26 ± 1.97%.

Table 2 shows the performance results for the testing dataset of 10 patient studies. The E-Net showed a mean DSC of 95.90 ± 1.56%, and the U-Net of 95.61 ± 1.82%. Additionally, we performed a comparison with the HRCT segmentations obtained using the region growing algorithm without any manual correction. Both DL algorithms outperformed the unsupervised region growing algorithm, which was incapable of correctly detecting many fibrotic regions, as reported in Section 2.1.

To test the differences between the results obtained with the DL algorithms, we compared the models using an analysis of variance (ANOVA) of the DSC computed for all patients’ studies. Results are summarized in Table 3. We observed that both methods obtained good performance in minimizing the difference between manual and automated segmentation, and they were significantly similar with respect to their provided outcome (a *p*-value > 0.05 indicates a strong similarity).

Nevertheless, E-Net and U-Net showed differences in their computational characteristics, as reported in Table 4. In particular, we reported a summary of parameters describing the computational complexity and performance of the DL models. E-Net appeared faster and more compact than U-Net due to the fact that it was developed with real-time applications in mind. Specifically, the E-Net model had an order of magnitude fewer parameters than U-Net. These characteristics had an effect on the size of disk required by the algorithm, ranging from about 6 MB required by E-Net to 65 MB required by the U-Net model (considering the 5 × 5 filter implementation). We estimated the time taken to complete a delineation using each of the DL models, by considering the average time required to obtain the output with each study. The time required by using a fairly advanced GPU device (NVIDIA QUADRO P4000, 8 GB VRAM, 1792 CUDA Cores) ranged from 20.32 s for E-Net to about 46.21 s for U-Net, if applied on a 3D dataset (average 600 slices of 512 × 512). Typically, such a dedicated hardware is not available, and computation is performed on a CPU. In order to test such a scenario, we used an Intel(R) Xeon(R) W-2125 CPU 4.00GHz processor. In this case, E-Net took an average of about 115 s to segment a dataset, while U-Net took about 1411 s. In addition, the E-Net algorithm had the peculiarity of making use of batch normalization layers, with not-trainable parameters and their gradients not backpropagated during training. It is worth noting that the reduced time and space required by the E-Net model to calculate a prediction makes it more compatible with clinical workflows installed on simple hardware.

**Figure 3:**
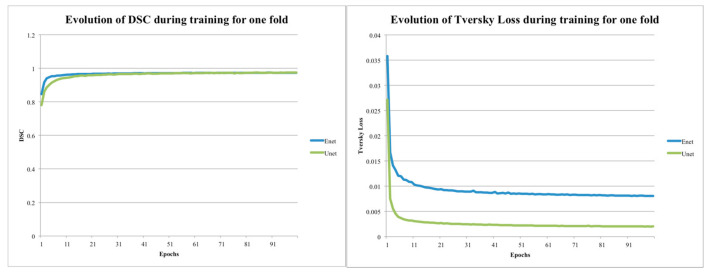
Training DSC and loss function Tversky loss plots for U-NET (5 × 5) and E-NET.

When the training phase of the algorithm was considered, we observed that also in this context, the E-Net model outperformed U-Net. In Figure 3, the training DSC and Tversky loss function of the two models for one fold are shown. Both the variables indicated that the E-Net model converged faster than U-Net. Specifically, the E-Net model reached a training DSC of about 0.95 in less than 5 epochs, while U-Net reached the same DSC in 13 epochs, respectively. Then, with the aim of updating the trained model as long as new training data became available, the E-Net model was more suitable for timely upgrades. In addition, the evolution of the Tversky loss showed that the training loss of the U-Net model was lower than that of the E-Net. Based on these results, it can be concluded that, from several points of view, it is more advantageous to use the E-Net model instead of the U-Net model.

## 4. Discussion

In this study, we investigate operator-independent solutions to support clinicians with the segmentation of the parenchyma when analyzing HRTC images of lungs. Our aim is to obtain an efficient, fast, and compact algorithm to perform lung segmentation in IPF patient studies. The anatomical target segmentation is the first step of a radiomics workflow, and when the target segmentation is performed manually, data is characterized by high variability. In addition, especially in the case of HRCT studies of lungs, where the analysis of hundreds of slices is required, manual segmentation is very time-consuming. For this reason, in order to standardize and optimize the target delineation step, we searched for the most suitable method to obtain an efficient parenchyma segmentation on a dataset of IPF cases without any radiologist supervision, unlike our previous studies [7,8,9]. Since segmentation represents the first step to create a texture-based prediction model, in order to evaluate the efficiency of the tested algorithms, we also considered whether their accuracy and reproducibility levels would be beneficial to a radiomics workflow. Here we describe the selection of the most efficient segmentation method. The application of the proposed segmentation model in a complete radiomics workflow will be described in a forthcoming paper.

Two deep learning models were tested to assess their feasibility in terms of accuracy in segmentation results, time required to train and implement the algorithm, and hardware and data requirements. We considered U-Net, a model already applied in many biomedical image segmentation tasks [29], and E-Net [30], developed for segmentation tasks required in self driving car tools. To our knowledge, the latter model has never been applied to lung segmentation before. DL models are still not widely applied in biomedical image data analysis workflows, because they require an amount of labelled data necessary to fulfil the training and validation tasks that is in general not available in clinical practice. The application of the model E-Net goes in the direction of developing models able to efficiently run with a limited amount of training data. We tested all models with the same limited dataset to evaluate and compare their performance. We observed that both DL models achieved very good results even with a limited amount of training data. The goal of having lower computational requirements and/or fewer trainable parameters is studied a lot in artificial intelligence research, i.e., the one-shot learning approach [37], the general approach of pruning trained networks [38], and the development of new CNN architectures specific for training with fewer training examples [39]. For example, Zheng et al. [37] proposed a one-shot active learning method that eliminated the need for iterative sample selection and annotation. This method was evaluated using a fungus dataset (4 training and 80 test images), a histopathology dataset (84/80), and an MR dataset (10/10).

Considering the state-of-the-art of lung delineation algorithms, U-Net results (DSC = 95.02%) similar to ours were obtained using a training set of hundreds of manually segmented images [25]. In [24], 617 studies were used to train U-Net with deep convolutional neural network obtaining a DSC > 98% and an inference time less than 1 min for a volumetric CT scan with around 600 slices using GPU hardware (versus ~20 s for our E-Net implementation). Luís et al. [40] presented a new feature extractor that worked as the mask R-CNN through transfer learning. They minimized the number of images used by the CNN training step. In this way, the number of interactions performed by the network decreased, and a DSC of 96.60% was obtained with a segmentation time of 11 s (GPU hardware) for each CT study with an unknown number of slices but certainly much less than 600 slices of an HRCT study. In [41], an algorithm based on random forest, deep convolutional network, and multi-scale super-pixels was proposed for segmenting lungs with interstitial lung disease (IDL) using the ILDs database [42] with an average DSC of 96.45%. Khanna et al. [43] implemented the residual U-Net with a false-positive removal algorithm using a training set of 173 images from three publicly available benchmark datasets, namely LUNA, VESSEL12, and HUG-ILD. Specifically, they implemented a U-Net with residual block, to overcome the problem of performance degradation, and various data augmentation techniques to improve the generalization capability of the method, obtaining a DSC > 98.63% using the five-fold cross-validation technique. In the same way, we obtained similar results (DSC = 98.34%) with far fewer images. For the automatic recognition of lung lesions, an active shape model approach based on low rank theory was proposed, obtaining a DSC of 94.5% using a small dataset of 35 multi-slice spiral CT lung images [44]. Segmentation times were not reported. Finally, for the pulmonary nodule detection, methods in two recent studies have been proposed. In the first one [45], a 3D lung segmentation method based on spatial constraints was proposed to achieve segmentation of lung parenchyma tissue and acquisition of target regions. The simple linear iterative cluster method was used to construct a 2D CT image into a super-pixel image matrix, and the matrix was thinned to reduce the dimension of the matrix. Then the nodes between adjacent slices were connected to construct a 3D structure of the lungs. The proposed algorithm obtained, for the nodule detection, a DSC > 80%. In the second study [46], the bidirectional chain code was used to improve the segmentation in 180 scans, and the support vector machine classifier was used to avoid false inclusion of regions. The proposed method provided a DSC of 95.85% and an average computation time of 0.68 s per CT slice on CPUs (considering 600 slices, ~6.8 min versus our 2 min on CPU).

In our study, we only use 32 CT studies obtained using two different acquisition scanners (Philips and GE scanners with a matrix resolution of 720 × 720 and 672 × 672, respectively) for the training phase. In addition, we considered 10 patient studies not included during the training process as the testing set. We obtained optimal results despite the small number of patient studies using the Tversky loss function, a data augmentation technique, and the k-fold strategy. Data standardization and normalization steps were applied to avoid uncontrolled increases of the weights and to help the model to converge faster. In order to overcome the issue concerning the low number of data, we used a five-fold cross-validation strategy using 2D slices from all patient cases, and the overfitting was reduced by applying data augmentation techniques. As a result, E-Net and U-Net are statistically identical but computationally different. In fact, E-Net is much faster than U-Net. E-Net has been developed for real-time applications and is therefore smaller and faster than the U-Net model. The E-Net model has a lower order of magnitude of parameters than U-Net. Using GPU hardware, we found that the E-Net needs only 20.32 s for the lung segmentation compared to 46.21s for U-Net. Likewise, when computations are performed on CPUs, the size of the model has a significant impact on the performance of DL models, which respectively take about 115.08 s (less than 2 min) for E-NET and 1411.69 s (more than 23 min) for U-NET. As shown in Figure 3, E-Net converges much faster than U-Net. Additionally, the training Tversky loss of the U-Net model is much less compared to E-Net, indicating overfitting despite reducing the number of filters in each layer of the original U-Net model. It is clear that the utilization of 5 × 5 filters in our U-Net architecture implementation is more memory demanding than the 3 × 3 convolution filter in the original U-Net. In terms of DSC, we observed, on the first training/testing fold, that the differences between using 3 × 3 filters versus 5 × 5 filters were 97.51 ± 3.01% and 98.23 ± 2.33%, respectively. Although the results are close, the 5 × 5 version produces better results with less variance. In terms of computational complexity, the 3 × 3 version is obviously smaller, as shown in Table 4. However, even with 3 × 3 filters, the U-Net model has 1,946,338 total trainable parameters compared to E-Net with only 362,992 parameters. That is still a fairly significant difference of a factor greater than five times. For the above-mentioned motivations, though the DL models are statistically not different, the E-Net model seems to be the best solution, and a benefit can be achieved in the use of this model, warranting the investment a developer would make in including the proposed segmentation model into radiomics workflows. The proposed approach not only removes the operator-dependence limitations of many radiomics studies, but it also obtains optimal results (DSC = 95.90%) using a small training dataset. Considering that the quality of radiomics studies depends on the imaging characteristics as reconstruction kernels, resolution, image quality, etc., the degree of image standardization must be as high as possible. In the same way, DL approaches are very sensitive to different image features. For these reasons, we used the same mono-centric dataset of 42 patient studies used in our previous works [7,8,9], and we did not performed an external validation with a dataset with very different image features. In the case of external validation, large datasets of labelled images could be assembled by merging datasets provided by different healthcare institutions. Conversely, because our aim is to identify an automatic, accurate, and fast segmentation method of the parenchyma without any radiologist supervision for future radiomics studies, we calculated the algorithm performance on 10 patient studies not used at all during the k-fold cross-validation strategy. This is a limitation of the study. In addition, future work will focus on the use of different loss functions (i.e., cross-entropy, focal, dice, etc.) and data augmentation techniques to make thorough comparisons. Another limitation of our study concerned the Checklist for Artificial Intelligence in Medical Imaging (CLAIM) proposed in [47]. We respected almost all the points proposed by the checklist. Nevertheless, a few points were not satisfied, such as the above-mentioned external validation, the measurement of inter- and intra-variability, the methods for explain ability or interpretability (e.g., saliency maps), and the failure analysis of incorrectly classified cases.

Finally, although validation in large image datasets is mandatory, the clinical application of DL may revolutionize the way we diagnose lung diseases and open the way towards a clinical decision-support system for risk stratification and patient management.

## 5. Conclusions

To extract the parenchyma from HRCT images of patients with IPF, we demonstrated the feasibility and efficacy of two different DL approaches using (i) the Tversky loss function into the training process, (ii) a suitable data augmentation technique, and (iii) the k-fold strategy. Both DL models highlighted good segmentation accuracy with a DSC of about 96%, with differences related to the training time and data requirements; results showed that E-Net and U-Net were statistically identical but computationally different. E-Net, which has been developed for real-time applications, is much faster than U-Net. The clinical application of our study can improve not only the diagnosis of lung diseases but also the management of patients through appropriate radiomics studies, where accurate and reproducible segmentations are mandatory, to correctly identify the prediction model. Future works include implementations with other state-of-the-art deep learning methods (e.g., in [6,11,13,24,25]), to compare them to our results.

## Figures and Tables

**Table 1:** Performance results for the validation dataset obtained as the average of the results computed in each loop of the five-fold cross-validation strategy.

	Validation Dataset (32 Patient 5-Fold)					
	E-Net			U-Net		
	Mean	±std	±CI (95%)	Mean	±std	±CI (95%)
Sensitivity	98.97%	2.09%	0.72%	97.67%	3.44%	2.76%
PPV	97.97%	1.86%	0.64%	99.00%	1.10%	0.88%
DSC	98.34%	1.44%	0.50%	98.26%	1.97%	1.57%
VOE	3.23%	2.73%	0.95%	3.35%	3.73%	2.98%
VD	0.87%	2.62%	0.91%	3.35%	3.73%	2.98%
ASSD	1.42	1.68	1.34	1.29	1.81	1.45

**Table 2:** Performance results for the testing dataset (10 patient studies) obtained as the average of the results computed from each Net trained in the five folds, and using the region growing algorithm.

	Testing Dataset (10 Patients’ Studies)								
	E-Net			U-Net			Region Growing		
	Mean	±std	±CI (95%)	Mean	±std	±CI (95%)	Mean	±std	±CI (95%)
Sensitivity	93.56%	3.41%	0.95%	92.40%	4.20%	2.60%	98.58%	11.18%	2.57%
PPV	98.44%	0.95%	0.26%	99.00%	0.82%	0.51%	77.71%	9.47%	2.21%
DSC	95.90%	1.56%	0.43%	95.61%	1.82%	1.13%	86.91%	8.94%	2.03%
VOE	7.84%	2.91%	0.81%	8.36%	3.40%	2.11%	23.15%	11.55%	2.67%
VD	−4.89%	3.82%	1.06%	−6.71%	4.43%	2.75%	26.85%	14.42%	3.33%
ASSD	2.31	0.70	0.43	2.26	0.60	0.37	9.72	4.51	1.07

**Table 3:** ANOVA on the dice similarity coefficient (DSC) showed no statistical differences between segmentation methods.

ANOVA	F Value	F Critic Value	*p*-Value
E-Net vs U-Net	0.18392749	4.964602744	0.677111335

**Table 4:** Comparison of computational complexity and performance of the two deep learning (DL) models. For U-Net, both implementations based on 3 × 3 and 5 × 5 filters were considered.

Model Name	Number of Parameters		Size on Disk	Inference Times/Dataset	
	Trainable	Non-Trainable		CPU	GPU
E-Net	362,992	8352	5.8 MB	115.08 s	20.32 s
U-Net (3 × 3)	1,946,338	0	23.5 MB	1211.99 s	39.53 s
U-Net (5 × 5)	5,403,874	0	65.0 MB	1411.69 s	46.21 s
